# Hypoxia Increases the Tempo of Phage Resistance and Mutational Bottlenecking of *Pseudomonas aeruginosa*

**DOI:** 10.3389/fmicb.2022.905343

**Published:** 2022-08-01

**Authors:** Ashley R. Schumann, Andrew D. Sue, Dwayne R. Roach

**Affiliations:** ^1^Department of Biology, San Diego State University, San Diego, CA, United States; ^2^Viral Information Institute, San Diego State University, San Diego, CA, United States

**Keywords:** bacteriophages, hypoxia, oxygen, phage resistance, pyomelanin, phage therapy, abiotic, galU

## Abstract

Viruses that infect bacteria (i.e., phages) are abundant and widespread in the human body, and new anti-infective approaches such as phage therapy are essential for the future of effective medicine. Our understanding of microenvironmental factors such as tissue oxygen availability at the site of phage–bacteria interaction remains limited, and it is unknown whether evolved resistance is sculpted differentially under normoxia vs. hypoxia. We, therefore, analyzed the phage–bacteria interaction landscape *via* adsorption, one-step, time-kill dynamics, and genetic evolution under both normoxia and hypoxia. This revealed that adsorption of phages to *Pseudomonas aeruginosa* decreased under 14% environmental oxygen (i.e., hypoxia), but phage time-kill and one-step growth kinetics were not further influenced. Tracking the adaptation of *P. aeruginosa* to phages uncovered a higher frequency of phage resistance and constrained types of spontaneous mutation under hypoxia. Given the interest in developing phage therapies, developing our understanding of the phage–pathogen interaction under microenvironmental conditions resembling those in the body offers insight into possible strategies to overcome multidrug-resistant (MDR) bacteria.

## Introduction

Multidrug-resistant (MDR) disease-causing bacteria are one of the biggest threats to global public health and security today. The United States Centers for Disease Control and Prevention (CDC) estimate that antibiotic resistance causes over 2.8 million illnesses each year, leading to more than 35,000 deaths as a result, with many more deaths from conditions complicated by resistant infections (CDC, 2019). While we have made significant progress in improving antibiotic stewardship, the discovery and commercialization of novel classes of antibiotics have waned (Sommer et al., 2017). New antimicrobial strategies continue to emerge, which focus on satisfying several criteria for innovation and safety, such as identifying agents with novel mechanisms of action or combinatorial treatments. The main preventive and therapeutic approaches under investigation include nanoparticles, bacteriophages (phages), endolysins, antibodies, vaccines, virulence suppressors, and microbiota modifications (Sommer et al., 2017; Luong et al., 2020b; Burki, 2021).

Phage therapy is widely being reconsidered to treat bacterial infections with virulent (lytic) phages, alone or in combination with traditional antimicrobials, as a promising strategy to combat MDR infections (Pirnay et al., 2018; Luong et al., 2020b; Hatfull et al., 2022). Phages are naturally occurring viruses that infect both gram-positive and gram-negative bacteria. As such, they pose limited adverse health effects in eukaryotic cells in patients. A substantial volume of preclinical data and an increasing body of clinical evidence indicate the immense therapeutic potential across a wide range of infectious diseases. While phages are generally unaffected by mechanisms of antibiotic resistance, there remain several clinical challenges such as their viral nature make it difficult to standardize as a drug, the need to employ multiple phage strains to increase drug spectra, and the lack of understanding the pharmacological properties of phages. Another obstacle in developing effective phage therapies is the evolution of phage resistance. Predicting the future of bacterial phage resistance is difficult, especially for evolutionary processes that are influenced by numerous unknown factors. Still, these are requirements during drug development to assess the risk of resistance arising against a new phage candidate during preclinical development.

Phage susceptibility and possible resistance testing are typically first performed *in vitro* to predict the *in vivo* effectiveness of treatment and to help guide the phage choice, dose, and timing of administration. One factor that is often overlooked during *in vitro* testing is the oxygen level at the site of a bacterial infection within the patient. As a result, the majority of the time, *in vitro* experiments are performed under ambient (normoxic) conditions, which may not recapitulate disease manifestations and phage activity in the body. Most mammalian tissue cells and organs experience oxygen partial pressures equivalent to 0.5–7% O_2_ (i.e., physioxia), with the exception of O_2_ tension ranging from 4 to 14% in the lungs, the liver, and the kidneys (i.e., hypoxia) (Carreau et al., 2011; Gan and Ooi, 2020). Recent studies have suggested that hypoxia can significantly alter the state of a bacterial cell, including differential gene expression, defense activation, metabolic states, receptor expression, and subsequent disease progression (Schaible et al., 2017). Therefore, the phage treatment net effect depends on a number of factors, including infection and replication dynamics of the target bacteria under low oxygen conditions.

To focus on research and development of novel antimicrobials, the WHO published its list of pathogens for which new drugs are in dire need. Within this broad list, ESKAPE (*Enterococcus faecium*, *Staphylococcus aureus*, *Klebsiella pneumoniae*, *Acinetobacter baumannii*, *Pseudomonas aeruginosa*, and *Enterobacter* species) pathogens were designated as “priority status” (WHO, 2017). *P. aeruginosa* is a ubiquitously distributed opportunistic human pathogen commonly associated with severe respiratory infections in patients with compromised immune defenses. Patients with chronic or inherited lung diseases, such as bronchiectasis and cystic fibrosis (CF), are highly susceptible to persistent pulmonary infection, with episodic exacerbations requiring hospitalization and intravenous antibiotics, with a subsequent risk of selection for MDR pathogens (Malhotra et al., 2019). The plasticity and adaptability of the relatively large *P. aeruginosa* genome (∼6.5 Mbp), conferred by a large repertoire of metabolic and regulatory genes, are the key elements of the pathogen’s ability to chronically persist in the host and evade antimicrobial therapeutics (Moradali et al., 2017). Usually described as a bacterium that favors aerobic growth conditions, *P. aeruginosa* is a facultative anaerobe well adapted to proliferate in conditions of partial or total oxygen depletion (Worlitzsch et al., 2002; Schaible et al., 2012; Gil-Marques et al., 2018). Therefore, the microenvironment at the site of infection likely plays a vital role in determining the outcome of *P. aeruginosa* infection.

There is an incomplete understanding of interactions between phages and bacteria under conditions that resemble those present in the body, such as reduced oxygen availability, making the clinical outcome of phage therapy unpredictable. In this study, we explored the impact of “hypoxia” on the interaction between *P. aeruginosa* and its phages. We demonstrated that exposure to environmental hypoxia did not alter time-kill kinetics of *P. aeruginosa* phages. Hypoxia, however, promoted a higher frequency of phage resistance and constrained types of spontaneous mutations. Developing our understanding of the phage–pathogen interaction under conditions that resemble those present in humans will provide us with new strategies to improve phage therapy efficacy.

## Materials and Methods

### Strains and Growth Conditions

The *P. aeruginosa* strain K (PAK) was cultured at 37°C overnight in Luria-Bertani (LB) broth, Miller. LB supplemented with 1.5% agar allowed for solid medium growth when required. The myovirus PAK_P1 (Forti et al., 2018) was propagated and purified as previously described (Luong et al., 2020a). Briefly, phages were added to mid-log growing bacteria at a multiplicity of infection (MOI) 0.1 and incubated overnight. Phage lysaes were centrifugred at 8,000 × g and phage supernatants were steriliezed by 0.22 μm dead-end filtraiton. Phage titers are expressed in plaque-forming units (PFUs) per ml and were measured using the serial spot method on LB agar.

### Mild Hypoxia Growth Conditions

**FIGURE 1:**
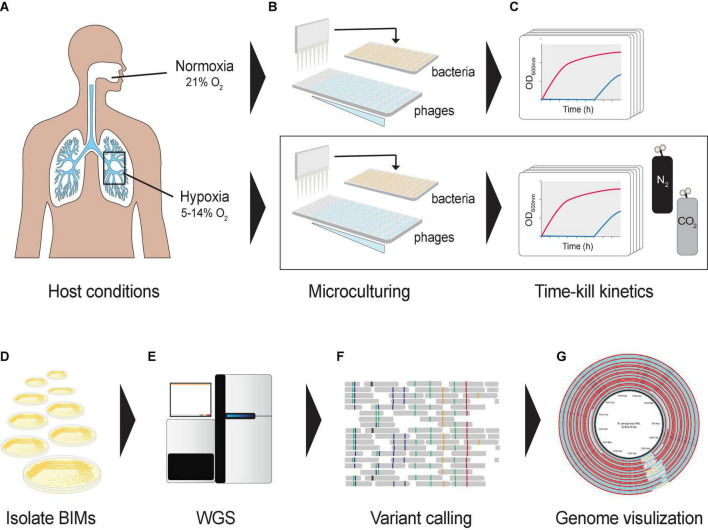
Graphical representation of the experimental design. **(A)** Oxygen levels in lungs mimicked using a hypoxic growth chamber. **(B)** A hypoxia culture-based assay was developed in a 96-well microtiter plate format to measure the phage–bacteria dynamics under different oxygen levels. Co-cultures were prepared in a hypoxic chamber fed with CO_2_ and N_2_. **(C)** Cultures were incubated in a microplate reader to assess bacterial optical densities at 600 nm under different oxygen levels. **(D)** Bacteriophage-insensitive mutants (BIMs) were isolated from cocultures **(E)** and their DNA extracted for whole-genome sequencing. **(F)** Detecting and characterizing sequence variation in BIM genomes, and **(G)** comparing evolution of multiple BIM genomes as a group against the central reference sequence.

Experiments were conducted under different oxygen levels (Figure 1). For hypoxic conditions (14% O_2_, 5% CO_2_) and ambient CO_2_-enriched (21% O_2_, 5% CO_2_) conditions, cultures were incubated in a hypoxic incubator chamber (Coy Laboratory Products, Grass Lake, MI, United States) before being transferred to a Clariostar microplate reader (BMG Labtech, Ortenberg, Germany) under the same conditioned atmosphere using compressed N_2_ and CO_2_ gases. For normoxic conditions with 21% O_2_ and 0.04% CO_2_, cultures were placed in a standard benchtop incubator (Eppendorf, Enfield, CT, United States) before transfer to the microplate reader. All media and buffers were preconditioned for >48 h in the test environment. Microplates were sealed with a BreathEasy™ membrane (Diversified Biotech, Dedham, MA, United States) to allow gas exchange.

### Time-Kill Kinetics

To evaluate the lytic activity of phage PAK_P1 under normoxic and hypoxic conditions, 2 × 10^6^ CFU of strain PAK was exposed to phages at MOIs 0.1, 1, and 10, and bacterial growth was monitored (OD_600_) at 37°C with an atmosphere controlled microplate reader. Media and buffers were preconditioned and microtiter plates were prepared in a hypoxic chamber. The area under the curve (AUC) was calculated Σ(Y_1_ + Y_2_)/2*(T_2_−T_1_). A total of five replicate microplates were performed and inoculated with different CFUs for each condition.

### Adsorption Constant

The phage adsorption rate was determined as previously described (Kropinski, 2009) under the aforementioned atmospheric conditions (Figure 1). Briefly, 1 ml of pre-warmed phage suspension was added to 9.0 ml of bacterial culture at 2 × 10^8^ CFU ml^–1^ at an MOI of 0.001 and incubated at 37°C with shaking at 120 rpm. About 50 μl of samples was diluted in 950 μl of fresh LB and 50 μl of chloroform (10% v/v) and placed on ice for 10 min before titering. The adsorption constant (*k*) was calculated (*k* = −slope/N), where N is the density of bacteria. A total of nine replicates were performed on different CFUs.

### One-Step Growth Curve

The one-step growth curves were determined by monitoring the dynamic changes in the number of phage particles during a lytic cycle (Hyman and Abedon, 2009). Briefly, phage suspension was mixed with 5 × 10^8^ CFU mid-exponential phase host cells to obtain MOI 0.1. After 6 min, the mixture was diluted 1,000 times in an LB broth to prevent infection by the new phage progeny and further incubated at 37°C. The results are the mean of three replicates ± standard deviation.

### DNA Extraction, Genome Sequencing, and Bioinformatics

Microtiter wells from time-kill curves were streaked onto LB agar to isolate bacteriophage-insensitive mutants (BIMs). Bacterial genomic DNA was extracted by NucleoSpin™ Microbial DNA Isolation Kit (Macherey-Nagel; Bethlehem, PA, United States), and sequencing libraries were prepared by MiGS (Pittsburg, PA, United States) using the Illumina Nextera Kit and then sequenced on the Illumina NextSeq 550 platform (San Diego, CA, United States). Raw 2 × 150 bp reads were trimmed by FASTP v.0.20.1^1^ and *de novo* assembled using SPAdes v.3.15.1 (Prjibelski et al., 2020), and reference guided assembled using MeDuSa v1.6 (Bosi et al., 2015) and the PAK reference genome (NC_002516.2). Annotation of a specific function of open reading frames (ORFs) was conducted using rapid annotations of subsystem technology (RAST v2.0) (Overbeek et al., 2014). Mutational analyses relative to ancestral PAK isolates grown under atmospheric conditions in the absence of phages were conducted using *breseq* v.0.35.5 (Deatherage and Barrick, 2014) and visualized using Circa software (OMG genomics).

### Statistics

Statistical analyses were performed using RStudio (Boston, United States) and visualized using the Jupyter Notebook. Tests used include the analysis of variance (ANOVA) and when required Dunnett’s and Tukey’s *post hoc* tests and Student’s *t*-test. A *p*-value of <0.05 was considered significant.

## Results

### Phage Time-Kill Dynamics Unaltered Under Hypoxia

**FIGURE 2:**
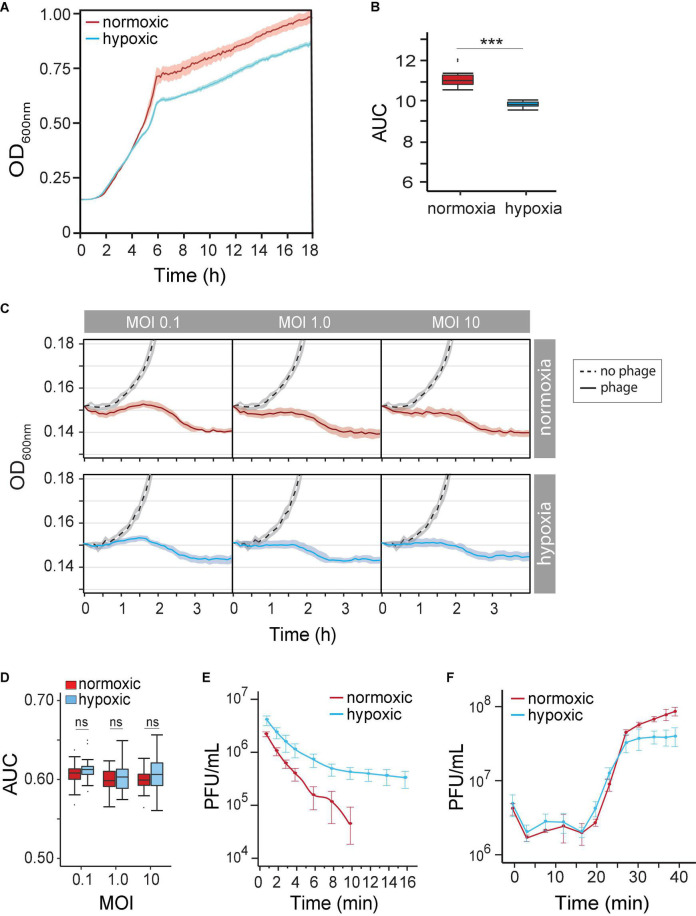
**(A)** *Pseudomonas aeruginosa* strain PAK population growth curves (based on OD at 600 nm) under normoxia versus hypoxia (14% O_2_/5% CO_2_) in the LB medium. **(B)** The difference in the *P. aeruginosa* strain K (PAK) population growth potential was measured as the area under the curve (AUC) over 18 h under normoxia (norm) and hypoxia (hypo). *p* < 0.001 **(C)** time-kill curves of no phage (dash line), phage under normoxia (red lines), and phage under hypoxia (blue lines) at different multiplicities of infection (MOIs). Microcultures were inoculated with 2 × 10^6^ CFU of bacteria. **(D)** AUC of time-kill kinetics over 4 h at different phage MOIs under normoxia (red) and hypoxia (blue). **(E)** The adsorption rate of phage PAK_P1 at MOI 0.001 under normoxia (red; *n* = 9) and hypoxia (blue; *n* = 9). **(F)** One-step growth curves of phage PAK_PI under normoxia (red circles) and hypoxia (blue circles). Phages were mixed with 5 × 10^8^ CFU bacteria at MOI 0.1. ***p < 0.001.

Our understanding of how the course of opportunistic bacterial infection is influenced by the microenvironment is limited. We first determined *P. aeruginosa* growth rate (μ_max_) and growth potential (AUC) under hypoxia (14% O_2_/5% CO_2_) and under normoxia (21% O_2_/0.5% CO_2_) in the absence of phages. We observed *P. aeruginosa* grown under both atmospheric conditions. As expected, however, decreased oxygen availability by 7% caused a 19% reduction in μmax (normoxia 0.091 OD_600 nm_⋅h^–1^ vs. hypoxia 0.074 OD_600 nm_⋅h^–1^) between 2 and 5 h (Figure 2A). Overtime, the reduced growth rate decreased the maximum OD_600 nm_ by 11% (OD_600 nm_ 0.958 ± 0.057 vs. 0.85 ± 0.02). Together, hypoxia caused a significant growth potential reduction of *P. aeruginosa* over 18 h at 37°C (AUC 10.38 ± 0.18 vs. 11.9 ± 0.58, *p* < 0.001) of 12.9% (Figure 2B). Nonetheless, changes in metabolism likely allow the pathogen to grow even under unfavorable conditions.

Because the physiological growth state of host bacterial cells may also influence the life cycle of phages, we next observed the phage’s lytic activity over time under hypoxia. Time-kill curves that monitor bacterial growth (and death) over a range of phage concentrations (i.e., MOIs) can evaluate the effect of hypoxia on phage lysis. However, the myovirus PAK_P1 time-kill kinetics of hypoxia-induced slower growth of *P. aeruginosa* was seemingly unchanged to that induced under normoxia (Figure 2C). In addition, bacterial growth potential (AUC) over 4 h was not significantly different between normoxia and hypoxia conditions (*p* = 0.17) after phage exposure (Figure 2D).

### Hypoxia Decreases Phage Adsorption Rate, Not Infection Latency

There are three stages of a lytic phage’s life cycle corresponding to the processes of adsorption, replication, and lysis. The rates and timing of these processes can thus be characterized as the phage’s life-history traits. Although the phage time-kill kinetics appeared not to be influenced by hypoxia, the phage–host interaction is a mass action, which assumes an equal influence of host cell density and phage adsorption rate (Shao and Wang, 2008). We sought to determine whether the altered state of the host under hypoxia influenced phage virion adsorption onto the surface of a susceptible host cell. Figure 2E shows a 66% decrease in phage PAK_P1 adsorption rate due to host cells grown under hypoxia (0.2288 vs. 0.0771 Log_10_PFU/ml min^–1^, *p* < 0.001). Because the influence of host density cell was controlled, this phenomenon might indicate a distinct effect on the bacterial outer membrane that delays phage binding under different oxygen levels.

However, we found that the timing of phage PAK_P1 replication and host lysis were similar. One-step growth curves allow us to define the timing of phage-induced host cell lysis (i.e., latent period), which is under the control of the phage gene products (e.g., holin). Phage PAK_P1 had a similar latent period of ∼14 min and a growth plateau at 30 min post-exposure (Figure 2F). Although one-step growth curves, which incorporate adsorption rate but avoid the effects of reinfections by phage progeny produced during replication, suggest that hypoxia did not affect phage generation time and the burst size, it was consistent with time-kill curves (Figure 2C).

### Hypoxia Raised the Rate of Phage Resistance

**FIGURE 3:**
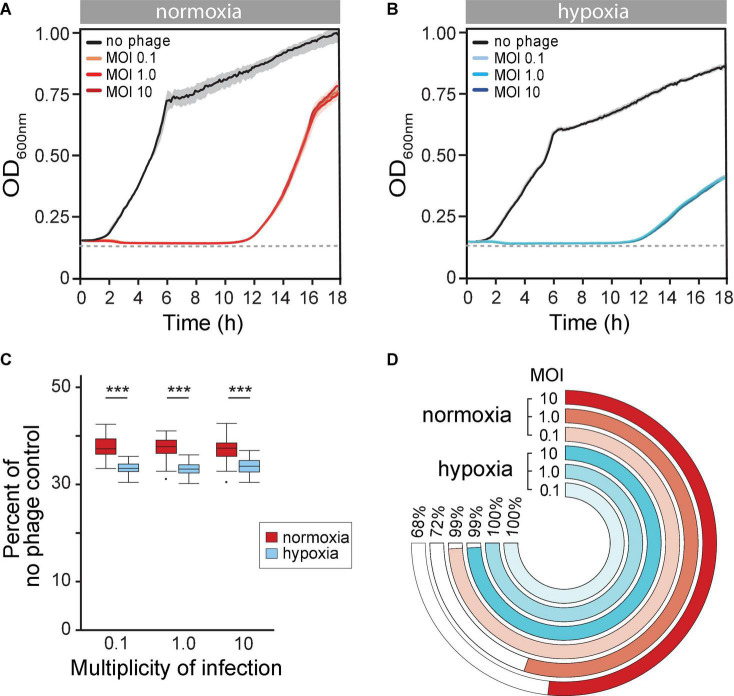
**(A,B)** Mean time-kill curves of phage PAK_P1 at different a multiplicity of infections (MOIs) under **(A)** normoxia and **(B)** hypoxia conditions. Microcultures under both atmospheric conditions inoculated with 2 × 10^6^ CFU and treated with phages at MOIs 0.1, 1.0, or 10 (*n* ≅ 42 per MOI per condition) showed gradual reductions to the limit of detection (dotted line) of bacterial density until bacteriophage-insensitive mutant (BIMs) outgrowth. **(C)** The phage time-kill area under the curve (AUC) for 18 h under normoxia (red) and hypoxia (blue) conditions normalized to no treatment control were significantly different (*p* < 0.001). **(D)** Percent of turbid microcultures 18 h after phage treatment at different MOIs. Treatment at MOI 0.1, 1.0, and 10 prevented bacterial growth under normoxia in 4 of 465, 209 of 741, and 119 of 372 microcultures, respectively. In contrast, bacteria growth was observed after 18 h under hypoxia in all 465 microcultures at each treatment MOI.

Next, we reasoned that the evolution of BIMs is perhaps inevitable *in vitro*. However, the altered state of host cells due to reduced O_2_ availability may also influence evolution phage resistance. We observed that the long-term phage time-kill kinetics were similar under both normoxia and hypoxia, with extended periods of controlled bacterial growth. There was, however, about a 1-h delay of bacterial regrowth that crossed the LOD under hypoxia (11.8 ± 0.61 vs. 10.56 ± 0.35 h, *p* = 0.002) (Figure 3A vs. Figure 3B). Furthermore, phage PAK_P1 appeared to have greater overall bacterial population control under hypoxia. The maximum rate of BIM growth (μmax) (i.e., bacterial regrowth) was reduced by 11% compared to BIM growth under normoxia, which also resulted in a lower OD_600max_ (Figure 3A vs. Figure 3B). In addition, we show in Figure 3C that phage treatment promoted a further 3% decrease in bacterial growth potential (AUC) compared to the exposure under ambient conditions after 18 h, normalized to bacterial growth in the absence of phages under the respective growth condition.

Although phages exhibited greater control of *P. aeruginosa* growth under hypoxia, we observed that nearly all phage-treated microcultures were turbid (>OD_600_ 0.3) after 18 h under hypoxia (Figure 3D, blue rings). This suggested that the hypoxia promoted a rising rate of phage resistance. In contrast, phage treatments at MOIs 0.1, 1.0, and 10 under normoxia were able to prevent regrowth in 1 (4 of 465), 28 (209 of 741), and 32% (119 of 372) of microcultures, respectively (Figure 3D, red rings). This implied that evolutionary dynamics were different under different atmospheric conditions, with high rates of phage resistance evolving quickly under hypoxia.

### Phage–Hypoxia Pressures Select for Increased Point Mutations

**FIGURE 4:**
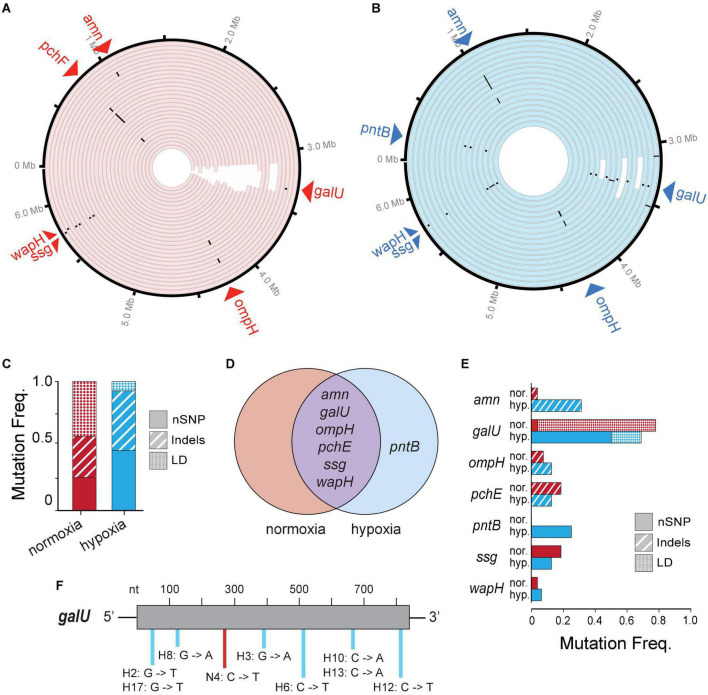
**(A,B)** A Circa plot showing spontaneous mutations in randomly isolated bacteriophage-insensitive mutants (BIMs) evolved under **(A)** normoxia (*n* = 27) and **(B)** hypoxia (*n* = 17) relative to no phage exposure. Both conditions exhibited mutations as non-synonymous single-nucleotide polymorphisms (nSNPs) (dots), insertion–deletions (dash), and large deletions (LD) (white rectangles). **(C)** Proportion of different mutation types identified in BIMs and **(D)** a Venn diagram showing the shared and unique genes with nSNPs. **(E)** Frequencies of BIMs shared mutated gene and frequency of gene mutation type. **(F)** Spectra of *galU* (UTP–glucose–1–phosphate uridylyltransferase) point mutations.

To look at genetic differences between evolutions of phage resistance under hypoxia compared to normoxia, we isolated 44 single BIM CFUs from 44 randomly selected individual time-kill cultures (i.e., wells) for whole-genome sequencing. We observed several types of genetic variants (or mutations) in the genome of each BIM isolated under either hypoxia or normoxia, including non-synonymous single-nucleotide polymorphisms (nSNPs), insertion–deletions (Indels), and large deletions (LD) (Figures 4A,B and Supplementary Table 1). The mean number of total mutations in each BIM was not significantly different between normoxia and hypoxia (1.30 ± 0.54 and 1.63 ± 0.62, respectively; *p* = 0.06). However, an evolution of resistance under hypoxia resulted in increased nSNPs (21%) and indels (14.9%), as well as a decrease in LDs (36%) compared to normoxia counterparts (Figure 4C). Thus, phage resistance evolution under normoxia resulted in a significant increase in large chromosomal deletions (85–438 kbp), which generally excised upward of 400 genes (Figures 4A,B and Supplementary Table 1). Interestingly, large chromosomal deletions of hundreds of genes were also observed in the evolution and diversification of *P. aeruginosa* associated with chronic infections in patients with CF (Hocquet et al., 2016).

Common spontaneous mutations after the evolution of phage resistance under either atmosphere include mutations in the *amn, galU, ompH, ssg*, and *wapH* genes (Figure 4D). Of these, *galU, ssg*, and *wapH* are each involved in lipopolysaccharide (LPS) biosynthesis, *ompH* encodes the TolC efflux pump, *amn* is involved in cellular respiration, and *pchE* encodes a siderophore. Because phages can use various cell surface structures to infect hosts, and bacteria often lose or modify these structures to gain resistance to phages (Labrie et al., 2010), phage PAK_P1 appears to bind LPS as its receptor. Under hypoxia, phage pressure was selected for the increased frequency of *amn* indels and *galU* nSNPs (Figure 4E). The latter is likely the result of opposing pressure against LD due to hypoxia. Point mutations occurred randomly throughout the *galU* gene (Figure 4F). This suggests that, while phage receptor mutations occur independent of the oxygen level tested, the variant type and frequency are correlated with the growth state of the bacterial host.

### Hypoxia Abolishes Pyomelanin Production

**FIGURE 5:**
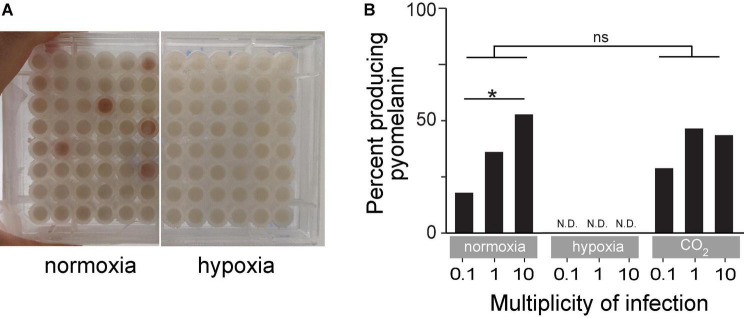
**(A)** An example of time-kill assay microplates after 18 h of incubation under normoxia or hypoxia. The reddish-brown pigmentation is pyomelanin. **(B)** Percent of turbid microcultures that also exhibited reddish-brown pigmentation under normoxia, hypoxia, or ambient CO_2_-enriched (21% O_2_/5% CO_2_) conditions. *p < 0.05, ns: not significant.

Several BIMs evolved under normoxia and displayed an easily observed reddish-brown pigment called pyomelanin (Figure 5A). Pyomelanin was produced in 17.9% of all phage-resistant microcultures at an MOI of 0.1 (*n* = 465), 36% at an MOI of 1 (*n* = 745), and 52.7% at an MOI of 10 (*n* = 372) under normoxia (Figure 5B). Melanogenic BIM genomes were identified to have LD (85–438 kbp), which attributed to the loss of both *hmgA* and the neighboring *galU*, among several other genes. Previous studies have shown that *hmgA* synthesizes homogentisate 1,2-dioxygenase, which is an essential component of the tyrosine catabolism pathway that converts homogentisic acid to 4-maleylacetoacetate (Hocquet et al., 2016). Mutation of *hmgA* causes an accumulation of homogentisic acid, which is secreted as pyomelanin. A total of three BIMs evolved under hypoxia (Figure 4B and Supplementary Table 1) with LDs that deleted *hmgA*, did not exhibit pyomelanin pigmentation (Figure 5A), even after further 24-h incubation under normoxia (data not shown).

## Discussion

Developing an understanding of the mechanisms underpinning the phage–bacteria interactions in both the broader context of parasite-host and predator-prey dynamics is key to improving the success of the phage therapy. In this study, we observed that the *P. aeruginosa* phage PAK_P1 lytic time-kill kinetics were largely indistinguishable under low-oxygen conditions compared to standard laboratory conditions. Our observations were consistent with our previous report (Mizuno et al., 2020) showing phages efficiently lysed *Citrobacter rodentium* under hypoxic conditions. We noted that reduced oxygen levels slowed phage adsorption to LPSs, suggesting that *P. aeruginosa*’s response to lower O_2_ was to alter its outer membrane. However, the slower growth rate of host cells under hypoxic conditions did not influence the phage PAK_P1 growth parameters (latent and burst size). Tracking the evolutionary dynamics within a bacterial population, as it adapted to phage selective pressures under low oxygen environments, we found that hypoxia promoted the evolution of phage resistance in nearly all phage–bacteria interactions compared to standard laboratory conditions. Among the mutants, we demonstrated that conserved *galU* mutation enabled the pathogen to adapt and persist, suggesting a reproducibility of evolution independent of the oxygen level. We also observed that hypoxic conditions switched the dominant genetic mutation type associated with resistance.

Phage replication is shaped by the cellular microenvironment; however, our current understanding of the influence underlying hypoxia-induced phage–bacteria dynamics is limited (reviewed in refs Hodges et al., 2021; Shareefdeen and Hynes, 2021). The local oxygen level can vary widely depending on the metabolic demand and blood supply of the tissue or organ (Carreau et al., 2011). While most *in vitro* studies on phage replication utilize cells cultured at atmospheric oxygen levels (∼21% O_2_), the vast majority of human tissues have oxygen levels much lower than this (referred to as hypoxia). Normal lungs have a partial pressure of oxygen (pO_2_) of between 42.8 and 110 mmHg (5.6–14% O_2_) and 5–9% CO_2_ (Le et al., 2006; Donovan et al., 2010). *P. aeruginosa* is preferentially an aerobic pathogen (Schaible et al., 2017), although we believe that *P. aeruginosa* could survive and grow under hypoxic stress to high cell densities, albeit at a reduced growth potential than under normoxic conditions. We observed that there was no significant decline in phage-induced lysis under mild hypoxia. However, mild hypoxia at 14% O_2_ may not recapitulate bacterial growth and phage activity inside the body. Mammalian tissue cells and organs experience oxygen partial pressures below 14% O_2_ (e.g., 0.5–14% O_2_) (Carreau et al., 2011; Gan and Ooi, 2020). Further limitations in oxygen could further alter the state of a bacterial cell, including decreasing gene expression, metabolism, and receptor expression. In our previous study, we indicated that lower oxygen hypoxia by 5% did not decrease phage lysis of *C. rodentium* in time-kill analysis at similar MOIs (Mizuno et al., 2020). We, however, found that a more extreme case of hypoxia caused a marked decrease in phage lytic activity at low MOIs (e.g., 0.01–0.001), which were not tested in the current study. Phage themselves are metabolically inert and their parasitism is largely dependent upon hijacking the host’s metabolic machinery and resources to replicate new virus particles. This is often deadly to host cells. One could hypothesize that facultative anaerobe cells under low oxygen possess an ample supply of energy metabolism to promote anabolism for the generation of macromolecules needed for virion replication and assembly.

Because time-kill kinetics provide insights over multiple phage infection cycles, we also characterized phage growth parameters, namely, adsorption constant, latent period, and burst size, under hypoxic and normoxic conditions. The lytic phage lifecycle begins with free-phage diffusion until attachment of the virion onto a specific host cell-surface structure. We found that 14% O_2_ significantly reduced phage PAK_P1 adsorption constant. The adsorption process is typically described by mass-action kinetics, which is assumed to be equally influenced by host density and adsorption rate (Shao and Wang, 2008). We demonstrated that phage PAK_P1 likely binds to the LPS in the outer membrane in *P. aeruginosa*. However, we cannot rule out hypoxia-induced alterations to LPS, which occur as a mechanism to evade recognition by mammalian defenses *in vivo* (Lam et al., 2011). The number of LPS monomers per bacterial cell appears to remain constant in different bacteria growth states (Walczak et al., 2012). On the contrary, one can assume that reduced bacterial motility under hypoxia might also influence phage adsorption (Schaible et al., 2017; Andre et al., 2022).

The latent period is defined by the timing of phage-induced host cell lysis, i.e., at the point when phage-progeny is released (Young, 2013). How phage PAK_P1 regulates lysis-timing remains to be determined, although we noticed that induced changes to the host cell under hypoxia did not affect the viral latent period and burst size. In contrast, several studies showed that the growth rate of bacteria influences phage growth parameters (Abedon et al., 2001; You et al., 2002; Golec et al., 2014; Nabergoj et al., 2018). A conclusion was that latent periods would tend to increase as host quality declines (Abedon et al., 2001). Our findings suggest that hypoxia did not diminish host quality to the extent of delayed lysis timing. Raya et al. (2011) showed that *Escherichia coli* under ambient and anaerobic growth conditions did not affect phage latent periods. This suggests that virulent phages precisely time the irreversible lysis of their host cells after infection independent of oxygen conditions.

The evolution of phage resistance was not influenced by the presence of the phage *per se* but rather the hypoxic conditions increased the adaptation tempo. Under normoxic conditions, phage PAK_P1 appeared to prevent bacterial growth in 25% of cocultures. In contrast, reducing O_2_ increased the pace of phage resistance, causing all phage–bacteria interactions to evolve resistance. Cellular metabolic rate is known to control mutation rates: mutations arise through unrepaired errors accrued during DNA replication and other damage-causing processes. Whole-genome sequencing determined that phage PAK_P1 was largely selected for mutations in genes related to LPS synthesis (*galU, ssg*, and *wapH*) under both normoxic and hypoxic conditions. For example, *galU* encodes a protein responsible for synthesizing UDP-glucose from UTP and glucose-1-phosphate during LPS outer core synthesis. Thus, modifications of the LPS core oligosaccharide were deemed the major mechanism for phage PAK_P1 resistance. The gram-negative bacterial LPS is a major component of the outer membrane that frequently plays a key role in pathogenesis. Bacterial adaptive changes including the modulation of LPS synthesis and structure are the conserved themes in infections, irrespective of the type of bacteria or the site of infection. In general, these changes result in immune system evasion, persisting inflammation and increased antimicrobial resistance (Priebe et al., 2004).

Populations of pathogenic bacteria undergo bottlenecks during infection due to host conditions such as those imposed by the host immune system and physical properties of tissues including oxygen availability (Levin et al., 1999; Common and Westra, 2019; Garoff et al., 2020). The evolution of phage resistance under hypoxia was selected overwhelmingly for point mutations in *galU*, whereas evolution under normoxia was selected for LD excising up to 8% of the genome, including *galU*. That is, bottlenecks and varying selection levels imposed by the phage and the oxygen level favor a subset of genetically encoded phenotypes in the population, leading to the loss of genetic variants. Contrary to our prediction, bottlenecking worked to the advantage of the bacterium providing fitness advantages under hypoxia. LD also excised *hmgA*, which caused the overproduction of the red-brown pigment pyomelanin. *hmgA* converts homogentisic acid to 4-maleylacetoacetate during tyrosine catabolism to break down pyomelanin. Pyomelanin can improve pathogen resistance to oxidative stresses and increase iron uptake, which may bolster survival in harsh environments (Rodriguez-Rojas et al., 2009). However, pyomelanin-producing variants only account for 20–30% of *P. aeruginosa* isolates from CF airways (Mayer-Hamblett et al., 2014). In an oxygen-rich environment under normal physiological conditions, the lung mucosal surface is susceptible to conditions of oxygen deficiency or tissue hypoxia in patients with CF (Page et al., 2021).

While novel insights were gained into how hypoxia influences phage–bacteria dynamics, this study has some limitations. Since this study examined phage resistance under mild hypoxia, examining the phage–bacteria interactions under severe hypoxia may reveal different dynamics and evolutionary patterns. For example, Ceyssens et al. (2010) found that, although phage PEV2 was able to infect *P. aeruginosa* growing anaerobically, the phage latent period was significantly longer. Moreover, the phage–bacteria dynamics can vary across phage species and different host species. Finally, all our experiments were performed in the LB medium, which is a nutritionally rich medium but not a defined medium that mimics the human–host environment.

Antimicrobial resistance represents one of the few crises that unite global interests, concerns for human and animal health, and agriculture. ESKAPE pathogens represent the paradigm for antibiotic resistance, pathogenesis, and disease transmission in both the community and clinical settings. It is now well recognized that phages have the therapeutic potential for the treatment and management of ESKAPE pathogens (Roach et al., 2017; Dedrick et al., 2019; Luong et al., 2020b; Cano et al., 2021; Johri et al., 2021; Lebeaux et al., 2021). To make accurate therapeutic decisions, it may be important to determine phage susceptibility and resistance development under clinically relevant oxygen conditions. However, predator-prey dynamic studies are mostly conducted under standard laboratory conditions, although this is rarely how phage–bacteria interact in nature. *P. aeruginosa* is present in many healthcare settings, especially in the context of chronic wounds, respiratory support, or urinary tract devices, where hypoxia predisposes for persistence, immune evasion, and antimicrobial resistance. Examining the phage–bacteria interactions under microenvironments similar to those found in the human body may lead to new therapeutic interventions.

## Data Availability Statement

The original contributions presented in the study are included in the article/Supplementary Material, further inquiries can be directed to the corresponding author.

## Author Contributions

ARS and ADS contributed to data acquisition. All authors contributed to the conception and design of the study, participated in data analysis and manuscript writing, read, and approved the final manuscript.

## Conflict of Interest

The authors declare that the research was conducted in the absence of any commercial or financial relationships that could be construed as a potential conflict of interest.

## Publisher’s Note

All claims expressed in this article are solely those of the authors and do not necessarily represent those of their affiliated organizations, or those of the publisher, the editors and the reviewers. Any product that may be evaluated in this article, or claim that may be made by its manufacturer, is not guaranteed or endorsed by the publisher.

## Footnotes

1
https://github.com/OpenGene/fastp
